# Investigation on the Effect of Persian Gum and Transglutaminase Enzyme on Some Physicochemical and Microstructural Characteristics of Low‐Fat Ultrafiltrated Iranian White Cheese

**DOI:** 10.1002/fsn3.4551

**Published:** 2024-10-24

**Authors:** Seyedeh Ameneh Habibi, Hossein Jooyandeh

**Affiliations:** ^1^ Department of Food Science and Technology, Agricultural Sciences and Natural Resources University of Khuzestan Mollasani Iran

**Keywords:** fatty acid profile, MTGase, PG, UF cheese

## Abstract

This study aimed to assess the effect of Persian gum (PG) and microbial transglutaminase enzyme (MTGase) on some physicochemical and microstructural characteristics of low‐fat ultrafiltrated Iranian white cheese during 60 days of cold storage. To manufacture the low‐fat cheese samples, PG (0%, 0.25%, and 0.5%) and MTGase (0, 0.5 and 1 U/g of protein) were used and cheeses (low‐ and full‐fat) without PG and MTGase were considered as controls. The obtained results revealed that the treated samples with PG had a higher titratable acidity, moisture content, and moisture‐to‐protein ratio (M:P), but a lower protein content as compared to the low‐fat control (*p* ≤ 0.05). Furthermore, the cheese samples treated with the higher level of MTGase (i.e., 1 U/g of protein) had a higher protein and fat content (*p* ≤ 0.05). Methyl palmitate (C_16:0_) and methyl oleate (C_18:1_) were found to be the most abundant fatty acids in the tested cheeses. Additionally, scanning electron micrographs revealed that addition of PG and MTGase improved the microstructure of the samples, so that the sample containing 0.5 U enzyme and 0.5% gum was more similar to its full‐fat counterpart and obtained the highest overall acceptance score among the cheeses. Therefore, our findings revealed that with incorporation of PG and MTGase, a low‐fat ultrafiltrated white cheese could be produced with an appropriate functional, physicochemical, and microstructural characteristics comparable to the full‐fat product.

## Graphical abstract

Incorporation of Persian gum (PG) and microbial transglutaminase enzyme (MTGase) caused a porous structure and microscopic waterholes in the low‐fat cheeses. Methyl palmitate (C_16:0_) and methyl oleate (C_18:1_) were found to be the most abundant fatty acids. Low‐fat containing 0.5 U enzyme (per g of milk protein) and 0.5% gum was more similar to its full‐fat counterpart and obtained the highest overall acceptance score among cheeses.

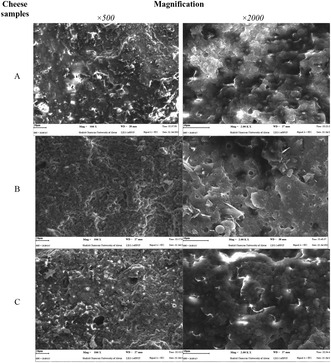

## 1 Introduction

Balanced nutrition is crucial for achieving and maintaining optimal health in individuals, and dairy products are an important source of obtaining essential nutrients. Dairy products have long been recognized and utilized for promoting human health and have been considered as an inseparable part of human diets for centuries. These products contain a variety of essential nutrients, such as protein and calcium, making them able to meaningfully improve the metabolic health and physical performance of the human body (Fox et al. 2017; Weaver 2021). Cheese is a highly popular milk‐based product enjoyed worldwide, primarily due to its appealing nutritional and sensory characteristics. The ultrafiltrated (UF)c white cheese manufactured using the ultrafiltration process is one of the widely popular and consumed cheese throughout the Middle East (Warsono et al. 2021). However, despite having a high nutritional value, the fat content of full‐fat cheeses often ranges from 25% to more than 45% on dry basis. Excessive dietary intake of fat is associated with an increased risk of various diseases, particularly obesity, coronary artery disease, type 2 diabetes, and colon cancer. Consequently, reduced fat cheeses like other low‐fat foods are growingly demanded by consumers. On the other hand, fat plays a key role in the mechanical behavior and sensory characteristics of different cheeses. Indeed, fat serves as a plasticizer within the casein matrix, decreasing the mechanical strength and softening the product (Danesh, Goudarzi, and Jooyandeh 2018; Wen et al. 2021). Additionally, free fatty acids generated through lipolysis are main flavor components of ripened cheeses (Jooyandeh, Kaur, and Minhas 2009).

Fat substitutes, also known as fat replacers, can be a potential solution for improving the mechanical behavior and sensorial characteristics of reduced fat foods. In fact, fat substitutes are a group of food ingredients that can mimic physical, rheological, and sensory characteristics similar to those of fat in different food, but offer meaningfully less calories. Gums are one of the most important fat substitutes in food products, which are also widely used in dairy industry (Akbari, Eskandari, and Davoudi 2019; Aires da Silva, Cristine Melo Aires, and da Silva Pena 2021; Wen et al. 2021; Jooyandeh, Saffari Samani et al. 2022). Gums are an important class of hydrocolloids. These polysaccharides are flexible biopolymers which are broadly utilized in food industry for their technological or nutritional purposes (Jooyandeh, Noshad, and Khamirian 2018; Saffari Samani, Jooyandeh, and Alizadeh Behbahani 2023; Zare‐Bavani, Rahmati‐Joneidabad, and Jooyandeh 2024). In the food sector, they are generally used in dairy products, baked goods, and beverages as thickening agent, stabilizer, or food coating/film and even for microencapsulation (Aires da Silva, Cristine Melo Aires, and da Silva Pena 2021). Persian gum (PG, also known as Zedo gum) is an anionic gum from the trunk and branches of the wild almond tree, *Amygdalus scoparia*, being mainly native to Iran. It is classified as a highly branched polysaccharide composed of galactose and arabinose as the main constituting monosaccharides with traces of rhamnose, mannose, and xylose (Sharif et al. 2021).

Furthermore, in the food industry, different enzymes are used to improve the technical characteristics, nutritional value, and production efficiency of various products (Kouravand et al. 2020; Seyed‐Moslemi et al. 2021; Jooyandeh, Momenzadeh et al. 2022). Transglutaminase is one of enzymes used in cheese‐making industries. Microbial transglutaminases (MTGases) have been utilized in industrial applications, since its initial production in 1989, using the *Streptomyces mobaraensis* bacterium through a conventional fermentation process (Warsono et al. 2021; Muhammad et al. 2021). Transglutaminases (protein glutamine gamma glutamyltransferase, EC 2.3.2.13) belong to the transferase family and catalyze the formation of inter‐ and intramolecular cross‐linkages between γ‐carboxamide groups of glutamine residues and ɛ‐amino groups of lysine residues in proteins, peptides, and primary amines. In fact, crosslinking of milk proteins with transglutaminase is considered as one of the most effective method to promote the functional properties of dairy products (Muhammad et al. 2021; Vasić, Knez, and Leitgeb 2023).

Various researches have been conducted to investigate the effect of MTGases and/or hydrochlorides on the cheese quality and quantity. Torabi, Jooyandeh, and Noshad (2021) reported that optimized symbiotic ultrafiltrated cheese treated with MTGase had the highest percentage of both saturated and unsaturated long‐chain fatty acids as compared with probiotic (without MTGase) and ordinary (without MTGase and inulin as prebiotic) control cheeses. This optimized symbiotic UF cheese had also a condensed texture and a higher texture score as compared to other cheeses during 60 days of cold storage period. Salunke et al. (2022a) conducted a study on the utilization of micellar casein concentrate and milk proteins treated with transglutaminase in various imitation cheese products. The researchers explored how MTGase treatment could potentially modify the surface properties of milk protein concentrate (MPC) and micellar casein concentrate, thereby improving the functionality of imitation cheeses, particularly mozzarella. Salunke et al. in other work (Salunke et al. 2022b) stated that MTGase treatment had a significant effect on modifying the stretch and melt abilities of MPC and micellar casein concentrate. Similarly, the research conducted by Monsalve‐Atencio et al. (2022) examined the impact of transglutaminase and its interaction with another enzyme called phospholipase on the composition, yield, texture, and microstructure of semisoft fresh cheese. The study found that the interaction of MTGase with phospholipase resulted in the highest moisture content in the cheese. This suggests that the combined use of MTGase and phospholipase could have economically beneficial applications in cheese making, potentially leading to improved yields and desirable textural characteristics in semisoft fresh cheese. Beirami, Hojjati, and Jooyandeh (2021) also evaluated the simultaneous effect of MTGase and PG on quality parameters of kefir during 30 days of cold storage. They reported that application of MTGase and PG enhanced the quality characteristics of the product.

PG is a cheap local gum produced in Iran, its annual production exceeds 700 metric tons, and about 200 to 400 tons of PG are exported annually to European and Arab countries with a low price (about 145 dollars per ton). PG as a new source of polysaccharides has drawn a great deal of consideration because of its medicinal (such as antiparasite and anticough agents) (Dabestani et al. 2018) and food applications (Jooyandeh et al. 2017). So far, no study has been assessed the coincident effect of MTGase and PG on the cheese quality. Therefore, this research was conducted to investigate the possible production of a low‐fat ultrafiltrated white cheese with an acceptable quality using an inexpensive local gum and MTGase.

## 2 Materials and Methods

### 2.1 Materials

PG was purchased from the local market (Mollasani, Khuzestan Province, Iran). After cleaning and separation of impure portion, the gum pieces were entirely grounded to a fine powder via a laboratory food processor and then sieved with 250 μm mesh size to attain extra fine particles. The cheese starter culture and commercial microbial rennet (Rennilase) were acquired from Chr. Hansen (Copenhagen, Denmark). The MTGase with the activity of 100 unit (U)/g of protein in a powder form was obtained from BDF Natural Ingredients (Girona, Spain). MPC comprise more than 70% protein, 16.50% lactose, 8% ash, and 1.30% fat procured from Pegah Dairy Company of Khorasan. Other used chemicals were purchased from Merck Company (Darmstadt, Germany).

### 2.2 Cheese Manufacturing

Ultrafiltrated cheese samples were produced in Pegah Dairy Company (Shush, Khuzestan province, Iran) as reported by Nosrati et al. (2023) with slight modifications. Fresh cow's milk of good quality was heated to 5°C and passed twice through bactofugation process to remove 99% of bacteria and then milk fat was standardized to 3% (w/w). Subsequently, the milk was pasteurized at 72°C for 15 s and then cooled down to about 5°C. To produce ultrafiltrated cheese, pasteurized milk was transferred to the UF cheese processing line, and the temperature of the milk reached to 50°C using a plate heat exchanger. Next, the milk was concentrated with an ultrafiltration (UF) system (up to 32 ± 0.28% dry matter content). The proximate composition of retentate was determined as 15.35% fat, 12.38% protein, 2.7% lactose, and 1.40% ash. To produce the low‐fat cheese samples, 50% volume of retentate was replaced with a MPC solution with the same retentate total solids TS (i.e., 32% TS). After adding PG in three levels (0%, 0.25%, and 0.50%, v/v), the mixture was homogenized at a pressure of 70 bar by the Ronghemachinary Homogenizer (JHG‐Q60‐P60, China), and pasteurized at 75°C for 15 s. Then, MTGase in the range of 0, 0.5, and 1 U/g of protein was added to the samples. The retentate was poured into 100 g plastic cheese containers, and 3% of the mixture of cheese starter cultures and rennet was injected to the retentate. Then, the packets were transferred to the coagulation tunnel at a temperature of 30.5 ± 0.5°C. After leaving the tunnel, the coagulated cheese samples were wrapped with parchment papers and 2% of salt (w/w) was added to the containers, and the packages were sealed with aluminum foil using automated crimp sealers. For cheese aging, the cheese samples were kept at 35°C–37°C, and after the pH dropped below pH 4.8, they were transported to the refrigerator at 6°C ± 1°C. All of the cheese samples were subjected to physicochemical and microstructural analysis after 1, 30, and 60 days of cold storage period.

### 2.3 Determination of Cheese Composition

Titratable acidity (T.A.) was evaluated by blending 9 g of the cheese sample with 9 mL of distilled water and titrating with 0.1 N sodium hydroxide using phenolphthalein (1% (w/v) in ethanol) as an indicator to an end point of stable faint pink color for 15 s. T.A. was expressed as a percentage of lactic acid. The total fat content was assessed according to the Gerber method (Jooyandeh and Minhas 2009). The moisture content of the cheese samples was determined by oven method at 110°C for around 2 h until a constant weight was reached (AOAC 2000). The Kjeldahl procedure was also utilized to determine the nitrogen content of the cheese samples (AOAC 2000). Using the total nitrogen content, the total crude protein content was assessed by multiplying the total nitrogen content by conversion factor of 6.38.

### 2.4 Fatty Acids Profile Analysis

#### 2.4.1 Lipid Extraction

Lipid extraction from cheese samples was performed in cold condition according to the method described by Torabi, Jooyandeh, and Noshad (2021). For this purpose, 10 g cheese was mixed with 100 mL of chloroform:methanol (2:1 v/v) solution, and the prepared mixture was homogenized (IKA Ultra‐TurraxT18 digital, Germany) for 4 min. Next, the prepared mixture was filtered by a filter paper (Whatman International Ltd., Grade 113, Maidstone, UK) and then 25 mL of saturated sodium chloride solution was added to the total filtrate. After separating the chloroform phase, the mixture was dehydrated over anhydrous sodium sulfate and concentrated using a rotary evaporator at 40°C under relative vacuum.

#### 2.4.2 Fatty Acids Methyl Esters Preparation

The fatty acid methyl esters (FAMEs) were prepared in accordance with Paszczyk and Łuczyńska (2020) with minor modification. Briefly, 15 drops of oil extracted from the cheese were transferred to a screw‐cap Pyrex test tube and 7 mL of hexane and 2 mL of methanolic potassium hydroxide (caustic potash) were then added to dissolve it. Next, the resulting mixture was stirred with a stirrer for 15 min, and then remained constant for 15 min until the fat was totally dissolved. After that time, 0.25 g of sodium hydrogen sulfate monohydrate was then dissolved in the mixture and centrifuged (5000 *g*, 15 min) at room temperature. Finally, the hexane layer containing the FAMEs was collected for gas chromatographic (GC) analysis.

#### 2.4.3 Determination of Fatty Acid Composition by Gas Chromatography

The chromatographic separation was carried out using a gas chromatograph (A‐Agilent 7890, the United States) equipped with a flame‐ionization detector (FID) and a capillary column with a length of 100 m, an internal diameter of 0.25 mm, and a thickness of 0.25 μm. The separation conditions were as follows: the sample injection volume was 2 μL, carrier gas was helium with gas flow rate 1 mL/min, the column temperature was adjusted from 100°C (for 1 min) to 260°C, ∆*t* = 15°C/min, and the injector temperature was 260°C. The amount of different fatty acids in the cheese samples were expressed as a weight percentage of total methyl esters of fatty acid and quantified based on peak areas (Paszczyk and Łuczyńska 2020).

### 2.5 Microstructural Analysis

The microstructures of cheese samples were observed with a scanning electron microscope (SEM; Leo 1455 VP, Cambridge, UK) operated at an accelerated voltage of 18.0 kV, following the method described by Razeghi and Yazdanpanah (2020). At first, the cheese samples were cut into 5 to 6 mm cubes and then stabilized by glutaraldehyde (2.5%, w/w) at 5°C for 3 h. Afterward, the cheese pieces were washed with distilled water and dehydrated in graded series of ethanol (40%, 55%, 70%, 85%, 90%, and 96%, 30 min for each series). The samples were defatted with chloroform (three times, each for 15 min), were covered with ethanol, and kept in a refrigerator until they were freeze‐fractured in liquid nitrogen into approximately 1 mm fragments. Finally, fractured samples were attached to the sample holder with silver paint and were evenly coated with a thin layer of gold for 6 min in a sputter coater (Balzers, type A450x; Baltek, Inc., Pfäffikon, Switzerland). The images were taken at 500× and 2000× magnification levels.

### 2.6 Overall Acceptance (Acceptability)

The acceptability of cheese samples was evaluated by a panel of 10 trained panelists. To ensure consistent temperature during the evaluation, the samples were allowed to reach room temperature for 30 min prior to the test (Razeghi and Yazdanpanah 2020).

### 2.7 Statistical Analysis

In this research, with regard to two examined variables (PG and MTGase, both at three levels), nine low‐fat cheese treatments were manufactured and evaluated at different storage periods (1, 30, and 60 days). The cheese sample without PG and MTGase was recognized as low‐fat control. To assess the main effects of variables, the obtained results were analyzed via completely randomized factorial design through SPSS software (SPSS, Inc., Chicago, 26th edition). Furthermore, to compare the nine low‐fat cheeses with full‐fat control sample, the one‐way analysis of variance (ANOVA) followed by Duncan's multiple range test (*p* ≤ 0.05) was employed. The graphs were created using the Excel 2016 software. All assays were performed in three replications (Mosallaie et al. 2020).

## 3 Results and Discussion

### 3.1 Titratable Acidity

**FIGURE 1:**
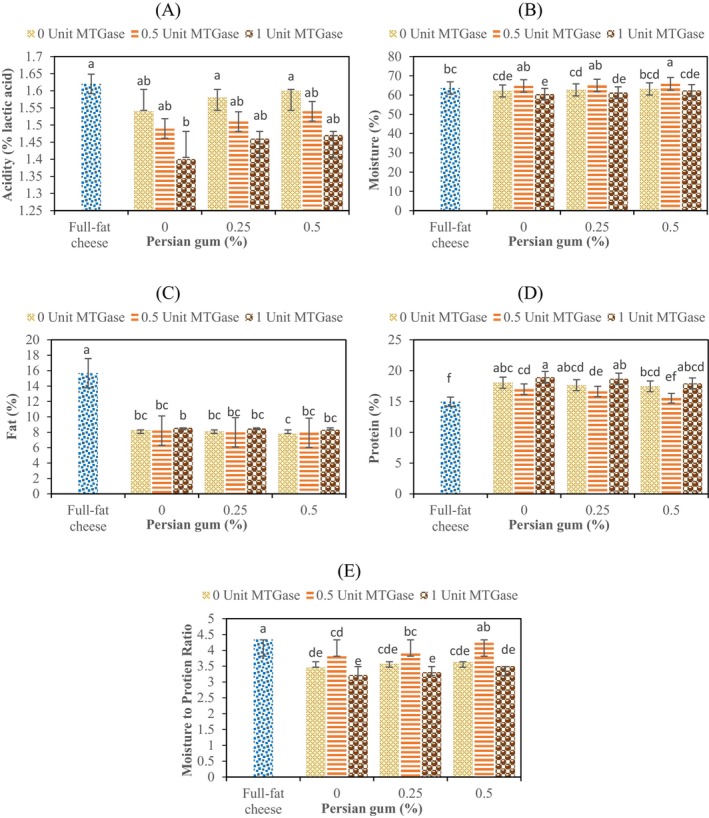
Effect of different concentrations of microbial transglutaminase (MTGase) and Persian gum (PG) on the physicochemical properties of low‐fat and full‐fat ultrafiltrated cheeses.

As illustrated in Figure 1A and Table 1, the acidity values of cheese samples were meaningfully affected by the examined variables. Generally, the samples treated with MTGase had a lower acidity compared to the control sample (*p* ≤ 0.05), while as the PG concentration increased, the T.A. slightly enhanced (*p* ≥ 0.05). Among the low‐fat samples, the samples containing 0.5% PG and without MTGase enzyme with 1.6% lactic acid had the highest T.A., and the treatments containing 0% gum and 1 U enzyme with 1.4% lactic acid had the lowest acidity. The reason for the decrease in acidity as a result of the addition of MTGase can be due to the decrease in the growth of the cheese starters. Peptides with low molecular weight or amino acids in milk are vital for the growth of *Streptococcus thermophilus*; these peptides are cross‐linked by the MTGase and become somewhat inaccessible to *Streptococcus* (Fox et al. 2004; Ozer et al. 2007). In agreement to our results, Ozer et al. (2007) indicated that by increasing the level of MTGase, the yogurt acidity was decreased, and this was referred to negative effect of MTGase on growth rate of lactic acid bacteria. Furthermore, the addition of PG resulted in a trivial increase in the acidity content of the treated samples. This may be attributed to the stimulation of the metabolic activity of the acid‐producing starter bacteria as a result of gum/polysaccharide addition (Fox et al. 2017). Portaghi et al. (2023) reported similar results and stated that the addition of basil seed and xanthan gum did not have a significant impact on the pH and acidity of cream cheese.

**TABLE 1:** The results of analysis of variance (ANOVA) of the effect of different concentrations of microbial transglutaminase (MTGase) and Persian gum (PG) on the physicochemical properties of cheese samples during 60 days of cold storage.

Parameter	Variable	Storage time (days)		
		1	30	60
Titratable acidity (% lactic acid)	MTGase concentration (U*)			
	0	1.50 ± 0.11^aB^	1.54 ± 0.14^aB^	1.68 ± 0.14^aA^
	0.50	1.45 ± 0.10^aB^	1.49 ± 0.14^aAB^	1.60 ± 0.16^aA^
	1	1.38 ± 0.15^aB^	1.44 ± 0.13^aAB^	1.53 ± 0.14^aA^
	PG concentration (%)			
	0	1.40 ± 0.15^aB^	1.46 ± 0.15^aAB^	1.57 ± 0.14^aA^
	0.25	1.45 ± 0.12^aB^	1.50 ± 0.10^aAB^	1.61 ± 0.15^aA^
	0.50	1.48 ± 0.12^aA^	1.51 ± 0.13^aA^	1.62 ± 0.18^aA^
Fat (%)	MTGase concentration (U)			
	0	8.03 ± 0.56^aAB^	8.42 ± 0.57^aA^	7.78 ± 0.62^bB^
	0.5	7.96 ± 0.63^aA^	8.20 ± 0.44^aA^	7.98 ± 0.54^abA^
	1	8.24 ± 0.45^aA^	8.56 ± 0.74^aA^	8.55 ± 0.70^aA^
	PG concentration (%)			
	0	8.16 ± 0.69^aA^	8.48 ± 0.45^aA^	8.42 ± 0.74^aA^
	0.25	8.06 ± 0.60^aA^	8.43 ± 0.79^aA^	8.09 ± 0.58^aA^
	0.50	8.01 ± 0.34^aA^	8.27 ± 0.54^aA^	7.79 ± 0.66^aA^
Moisture (%)	MTGase concentration (U)			
	0	64.92 ± 0.90^abA^	61.19 ± 0.97^bB^	61.88 ± 0.17^bB^
	0.5	65.45 ± 0.65^aA^	64.55 ± 1.03^aA^	65.28 ± 1.14^aA^
	1	64.65 ± 0.65^bA^	59.96 ± 1.19^cB^	59.36 ± 0.94^cB^
	PG concentration (%)			
	0	64.69 ± 0.86^aA^	61.27 ± 2.30^bB^	61.35 ± 3.01^bB^
	0.25	65.06 ± 0.82^aA^	61.88 ± 2.08^abB^	61.89 ± 3.09^bB^
	0.50	65.27 ± 0.70^aA^	62.54 ± 2.37^aB^	63.29 ± 2.32^aB^
Total protein (%)	MTGase concentration (U)			
	0	16.50 ± 0.60^aB^	18.51 ± 0.59^bA^	18.16 ± 0.40^bA^
	0.5	15.40 ± 1.50^bB^	16.89 ± 0.65^cA^	16.66 ± 0.67^cA^
	1	16.84 ± 067^aB^	19.16 ± 0.49^aA^	19.50 ± 0.90^aA^
	PG concentration (%)			
	0	16.73 ± 0.65^aB^	18.62 ± 1.05^aA^	18.63 ± 1.203^aA^
	0.25	16.50 ± 0.64^aB^	18.18 ± 1.06^abA^	18.28 ± 1.51^aA^
	0.50	15.51 ± 0.49^bB^	17.76 ± 1.19^bA^	17.41 ± 1.17^bA^
Moisture:protein ratio	MTGase concentration (U)			
	0	3.94 ± 0.14^bA^	3.31 ± 0.13^bB^	3.41 ± 0.11^bB^
	0.5	4.21 ± 0.34^aA^	3.83 ± 0.16^aB^	3.93 ± 0.20^aB^
	1	3.84 ± 0.17^bA^	3.13 ± 0.13^cB^	3.05 ± 0.24^cB^

	PG concentration (%)			
	0	3.87 ± 0.18^bA^	3.31 ± 0.30^cB^	3.31 ± 0.37^bB^
	0.25	3.95 ± 0.16^bA^	3.42 ± 0.31^bB^	3.42 ± 0.45^bB^
0.50	4.17 ± 0.37^aA^	3.54 ± 0.36^aB^	3.66 ± 0.37^aB^

*Note:* Different small letters in each column (treatments, i.e., MTGase or PG levels) and different capital letters in each row (storage days) indicate a significant difference at 95% level.

* U: unit per g protein in cheese milk.

Furthermore, the full‐fat control sample had the highest acidity as compare with low‐fat cheeses. As shown in Figure 1B,C, the full‐fat cheese had the highest fat and a considerable amount of moisture content. As the cheese fat content increases, the amount of free fatty acids through lipolysis enhances and this result in a higher T.A. (Jooyandeh, Kaur, and Minhas 2009). Also, the higher amount of cheese moisture enhances the amount of soluble chymosin and the ratio of soluble protein to total protein, resulting in an increase in acidic carboxyl groups and an intensification in the conversion of lactose to lactic acid (Rahimi et al. 2007; Rostamabadi, Jooyandeh, and Hojjati 2017).

Moreover, the storage time during refrigerated conditions had a significant effect (*p* ≤ 0.01) on the values of T.A. (Table 1). The acidity values of all samples increased throughout the storage period; the mean acidity values of cheese samples increased from 1.44% to 1.60% lactic acid between the beginning and the end of storage period. In similar results, an increase in acidity during storage time have been reported for different fermented dairy products such as white cheese (Eljagmani, Altuner, and Yildiz 2020), kefir (Beirami, Hojjati, and Jooyandeh 2021), and yogurt (Jooyandeh and Alizadeh Behbahani 2024). This is mainly attributed to starter culture activity; since it maintains its original biochemical reactions during storage for prolonged periods (Temiz and Çakmak 2018).

### 3.2 Moisture Content

Moisture is an important factor for cheese, because it directly influences the texture and other quality characteristics of the product. It also serves as reactant in various biochemical and enzymatic reactions and is bounded with the protein to maintain elasticity of cheese (Murtaza et al. 2017). As can be seen in Figure 1B, the addition of gum and enzyme resulted in significant changes in the moisture content of the samples (*p* ≤ 0.05). The highest amount of moisture (65.81%) was related to low‐fat cheese sample containing 0.5 U MTGase and 0.5% PG, while the lowest moisture content (60.45%) was observed for the sample with 1 U MTGase and 0% PG. Changes in the moisture content of cheese samples during the storage period are presented in Table 1. In general, the moisture content decreased significantly until the 30th day of storage period and then showed an increasing trend until the end of the period. Moisture changes are indeed important physical processes happening with food during the storage time. Both dry and moist foods can be affected by moisture loss or gain, which can have significant impacts on the characteristics of the food. Similarly, the study conducted by Abou‐Soliman, Awad, and El‐Sayed (2020) found that the moisture content of cheese samples treated with MTGase was significantly higher than control cheese (without MTGase). According to the researchers, the cross‐linking induced by MTGase increased the free volume within the curd matrix and resulted in a finer protein network, allowing the curd to retain more water.

### 3.3 Total Fat Content

Fat plays a significant role in cheese yield due to formation of stable fat–protein network within the cheese matrix. Lowering the fat content deteriorates the cheese protein network and reduces the cheese yield. The hydrocolloids, such as certain gums or polysaccharides, can directly bind the water and interfere with the shrinkage of the casein matrix. This lowers the driving force involved in expelling water from curd particles and consequently improves the yield (Murtaza et al. 2017). The changes in the fat content of ultrafiltrated cheese samples due to the addition of MTGase and PG are shown in Figure 1C. Results revealed that addition of PG slightly reduced the fat content of UF cheeses, possibly because of an increase in cheese moisture. Gums as fat substitutes have the ability to increase the moisture content of low‐fat cheeses (Torabi, Jooyandeh, and Noshad 2021; Jooyandeh 2024). Romeih et al. (2002) in a similar results revealed that low‐fat white‐brined cheeses prepared with hydrocolloids had a higher moisture content. Furthermore, treatment with MTGase had a mutual effect on the cheese fat of the samples. Treatment with 0.5 U/g MTGase generally caused a slight decrease in the cheese fat, while treatment at the higher MTGase concentration (i.e., 1 U/g protein) significantly decreased the cheese fat content. These changes similarly seem related to the effect of the variables on the cheese moisture.

The MTGase enzyme at a lower concentration moderately stabilizes the milk protein gel matrices by forming cross intra‐ and extra linkages between isopeptide bonds of ε‐(γ‐glutamyl)‐lysine and consequently increases the water‐holding capacity of the casein network. However, at a higher MTGase concentration, a compact cheese texture is produced due to whey expulsion as a result of extra linkages (Danesh, Goudarzi, and Jooyandeh 2018; Jooyandeh 2024). The highest amount of fat was measured in the full‐fat cheeses (15.67%), whereas the lowest amount of fat (7.82%) was perceived in low‐fat sample containing 0 U MTGase and 0.5% PG (*p* ≤ 0.05). As presented in Table 1, the results of ANOVA revealed that the fat content of cheese samples was increased until the 30th day of the cold storage and then decreased up to the end of the storage period (*p* ≤ 0.05). The increase in the fat content during the first month of storage can be attributed to the reduction of cheese moisture content, which increases the other cheese constituents. Additionally, with the increase of storage time (30th day up to the end of 60 days of storage), the fat content decreased due to the development of several biochemical changes, such as breaking down of fat through cheese lipolysis and proteolysis which cause an increase in the moisture content (Nosrati et al. 2023). In similar results, Torabi, Jooyandeh, and Noshad (2021) reported a decrease in fat content at the end of the storage period due to the presence of lipolytic enzymes (particularly produced by lactic acid bacteria) and the minor increase in the cheese moisture.

### 3.4 Total Protein Content and M:P Ratio

The results obtained from the effect of two independent variables, that is, MTGase and PG, on the total protein content and M:P ratio of tested cheese samples are presented in Figure 1D,E, respectively. As it shown in Figure 1D, there are significant differences in the total protein content and M:P ratio of the cheese samples (*p* ≤ 0.05). Minimum and maximum amounts of protein in the cheese samples were determined in the full‐fat cheese sample (14.96%) and the sample containing 1 unit enzyme and 0% gum (18.92%), respectively (*p* ≤ 0.05). In contrast, the lowest and the highest amounts of M:P ratio were measured in the samples containing 1 unit enzyme and 0% gum (3.22) and the full‐fat cheese control (4.34), respectively (Figure 1E). Even though decreasing the amount of fat caused a significant increase in the protein content of the cheese samples, as it clears in Figure 1D, no significant difference was found between full‐fat control and low‐fat cheese containing 0.5% PG and 0.5 U MTGase (4.34 vs. 4.24). Therefore, this UF white sample cheese could be regarded as the best and optimized low‐fat cheese. Our finding was similar with those reported by Jooyandeh et al. (2017), who observed that reducing the fat content in Iranian white cheese containing Persian and almond gums resulted in a significant increase in M:P ratio and a noticeable decrease in protein levels. Furthermore, Danesh, Goudarzi, and Jooyandeh (2018) exhibited that treatment of milk with TG increases the M:P ratio of the produced low‐fat Iranian white cheeses causing a significant reduction in fracture stress, storage modulus, and Young's modulus.

Results also revealed that during the cold storage period, the protein content of the cheese samples at the first month of storage significantly increased (*p* ≤ 0.05), while thereafter slightly decreased (*p* > 0.05) till the end of 60 days of storage period. The mean values of cheese protein at the beginning, middle, and the end of storage period were determined as 16.25%, 18.19%, and 18.11%, respectively. The increase in protein content up to day 30 of the storage period can be associated with the reduction of cheese moisture, while the slight decrease in protein content at the end of the storage period could be attributed to proteolysis and the conversion of proteins into peptides and amino acids (Jooyandeh 2024). In agreement to our findings, Torabi, Jooyandeh, and Noshad (2021) reported that fat and protein contents were meaningfully increased until the 30th day of storage, followed by a subsequent decrease.

### 3.5 Fatty Acid Composition

Foods contain various types of saturated fatty acids (SFAs) and unsaturated fatty acids (USFAs), each have different effects on lipoprotein metabolism. SFAs can affect the risk of development of numerous diseases. The consumption of milk and dairy products, which naturally contain high levels of SFAs, has been associated with adverse health effects and the development of various diseases, including cardiovascular disease, type 2 diabetes mellitus, obesity, and certain types of cancer. However, recent research findings have suggested that the link between SFAs and the incidence of these diseases may be less straightforward than previously assumed (Kawęcka, Radkowska, and Sikora 2020; Paszczyk and Łuczyńska 2020).

Table 2 shows the comparison between the fatty acids composition of full‐ and low‐fat cheese controls with optimized low‐fat cheese. The results indicated that SFAs are the predominant type of fatty acids found in the fat extracted from the all cheese samples. Methyl palmitate (C_16_:0) and methyl oleate (C_18_:1) were found to be the main fatty acids in the UF cheeses. Among the cheeses, full‐fat control cheese had a highest SFAs and USFAs content. The amounts of SFAs and USFAs in the full‐fat control were determined as 131.78 and 45.50 g/100 kg cheese, while these values for low‐fat control were 67.53 and 25.62 g/100 kg cheese, respectively. Treatment of milk for cheese making by PG (0.5%) and MTGase (0.5 U/g protein) enhanced the fatty acids content of the low‐fat cheese and it resembled to its full‐fat counterpart. The amounts of SFAs and USFAs in optimized cheese sample were recorded as 101.69 and 36.06 g/100 kg cheese, respectively.

**TABLE 2:** Fatty acid profile (FAP) of ultrafiltrated Iranian white cheeses (g/100 kg cheese) at the end of 60 days of storage at refrigerator temperature.

Fatty acids	Full‐fat control	Low‐fat control	Optimized low‐fat cheese (0.5% PG and 0.5 U MTGase)
C4: 0	16.13	8.56	12.45
C6: 0	11.12	5.02	8.92
C8: 0	7.14	4.00	5.95
C10: 0	8.18	4.16	5.98
C12: 0	9.29	4.98	6.73
C14: 0	15.23	8.94	11.61
C16: 0	36.13	17.18	27.73
C18: 0	10.12	5.72	7.65
Other SFAs	18.44	8.97	14.67
Total SFAs	131.78	67.53	101.69
C18:1	30.01	17.35	24.66
C18:2	2.98	1.76	2.15
C18:3	1.11	0.64	0.83
Other USFAs	11.40	5.87	8.42
Total USFAs	45.50	25.62	36.06
Total fatty acids	177.28	93.15	137.75

Abbreviations: MTGase, microbial transglutaminase; PG, Persian gum; SFA, saturated fatty acids; USFAs, unsaturated fatty acids.

Similarly, Paszczyk and Łuczyńska (2020) stated that SFAs were predominant in the fat extracted from cow, sheep, and goat cheeses. Additionally, palmitic acid (C_16_:0) was the major SFA and oleic acid (C_18_:1 cis9) was identified as the predominant USFA. In another study, Torabi, Jooyandeh, and Noshad (2021) reported that symbiotic UF cheese (treated with MTGase and inulin) significantly had different fatty acids profile as compared to control cheese samples. Among the different cheese samples, the optimized symbiotic ultrafiltrated cheese had the highest percentage of both saturated and unsaturated long‐chain fatty acids.

### 3.6 Microstructural Characteristics

**FIGURE 2:**
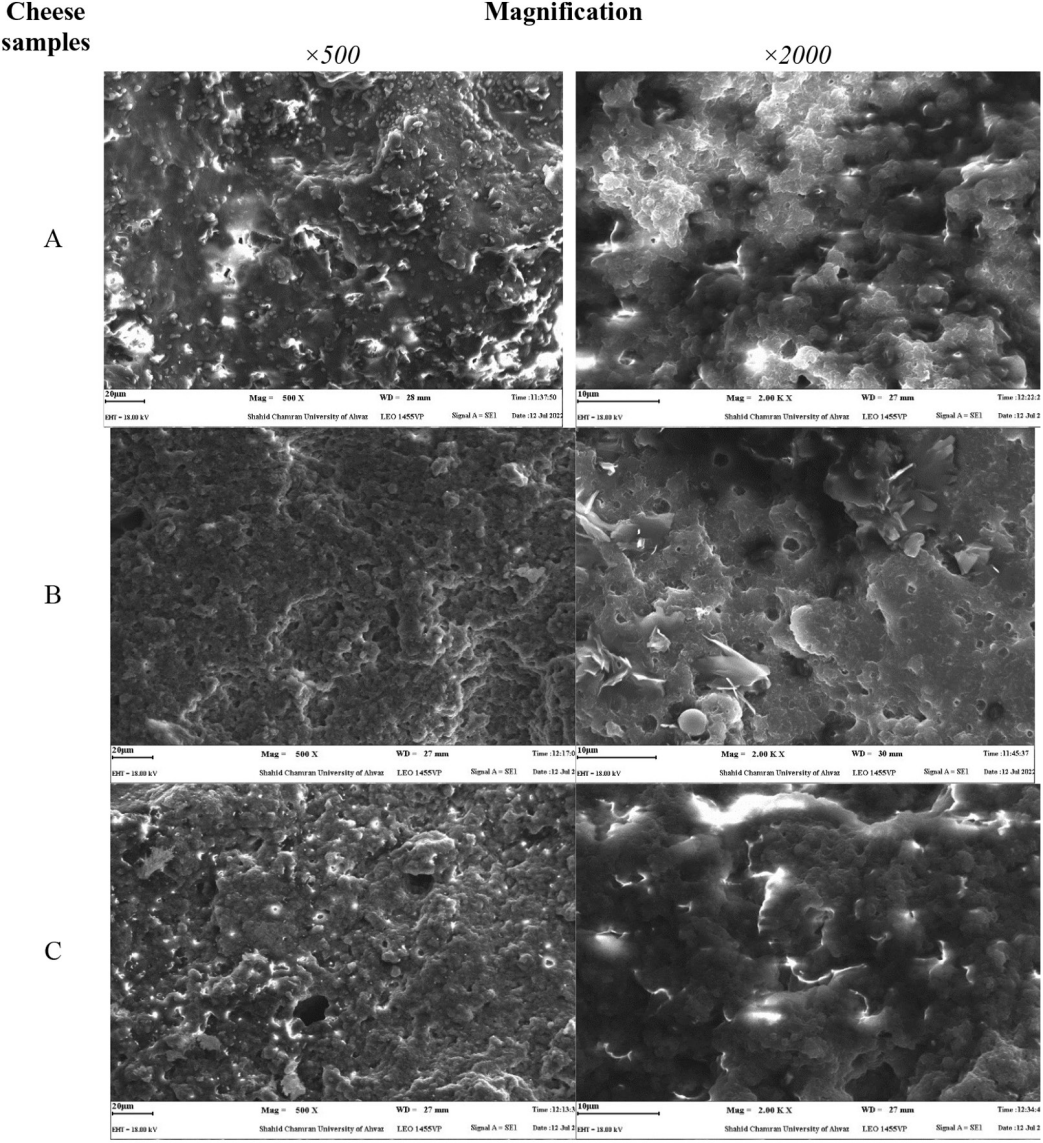
Scanning electron microscope (SEM) micrographs of ultrafiltrated cheeses: (A) control full‐fat, (B) control low‐fat, and (C) the optimized samples containing 0.5% gum and 0.5 U enzyme.

The scanning electron micrographs of the control (low‐ and full‐fat) and optimized cheeses are presented in Figure 2. In general, the low‐fat control as compared to full‐fat cheese had a compressed and rigid structure and showed less porous characteristic. Many researchers denoted that fat acts as a softener or lubricant in cheese and other food protein gels (Wen et al. 2021; Jooyandeh et al. 2017). However, treated sample with MTGase and PG had a porous structure and resembled to the full‐fat cheese. As a result, an appropriate amount of enzyme and gum could improve the sensory and mechanical characteristics of the UF cheeses. The porous structure and microscopic waterholes of optimized sample is probably due to an augmented hydration capacity of casein network (Danesh, Goudarzi, and Jooyandeh 2018; Muhammad et al. 2021). In similar study, Cadavid et al. (2020) investigated the impact of MTGase on the functional characteristics of quark‐type cheese. The findings of this study revealed that MTGase can improve the cheese microstructure through modifying the protein accumulation patterns and proper formation of three‐dimensional casein network.

### 3.7 Overall Acceptance (Acceptability)

In order to maintain the cheese freshness, extending the shelf life of the product is an important issue. In addition, sensory attributes play a significant role in cheese quality and determine market demand. The preferences and palates of people in evaluating the sensory quality of cheeses may differ from one to another, which often depends on people's familiarity with the types of cheeses in that particular market. Iranian ultrafiltrated white cheese is a popular kind of soft cheese, and it is consumed as the main part of traditional Iranian breakfast (Warsono et al. 2021).

**FIGURE 3:**
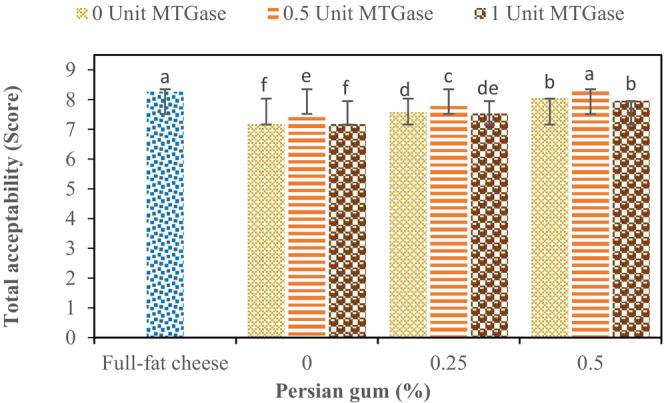
Effect of different concentration of microbial transglutaminase (MTGase) and Persian gum (PG) on the overall acceptance of ultrafiltrated cheese samples.

The findings of the study indicate that all of the independent variables had a significant impact on the overall acceptance of the cheese samples (*p* ≤ 0.05). Although low‐fat cheese control had significantly lower score (7.17) than full‐fat one (8.26), incorporation of PG and MTGase improved the cheese acceptance (Figure 3). Results of data analysis revealed that with increasing gum concentration, the overall acceptance of the samples increases significantly (*p* ≤ 0.05). Furthermore, by increasing the concentration of MTGase up to 0.5 U/g protein, the overall acceptance score increased, but further increasing the enzyme concentration up to 1 U/g protein resulted in a decrease in the acceptance rate. The highest overall acceptance score (8.27 of 9) was obtained in the optimized sample containing 0.5% PG and 0.5 U MTGase. In addition, as it shown in Table 3, by passing the storage time, the cheese acceptability increased; but these changes were not significant (*p* > 0.05). The increase in cheese acceptability during the storage is due to biochemical changes in the cheeses, particularly lipolysis and proteolysis which cause liberation of flavor precursors, that is, free fatty acids and amino acids.

**TABLE 3:** The results of analysis of variance (ANOVA) of the effect of different concentrations of microbial transglutaminase (MTGase) and Persian gum (PG) on the overall acceptance of UF low‐fat cheese samples during the cold storage.

Parameter	Factor	Storage time (days)		
		1	30	60
Overall acceptance (score)	MTGase concentration (Unit/g protein)			
	0	7.49 ± 0.40^b^	7.58 ± 0.35^b^	7.73 ± 0.44^b^
	0.50	7.73 ± 0.41^a^	7.82 ± 0.36^a^	7.92 ± 0.39^a^
	1	7.53 ± 0.39^b^	7.55 ± 0.34^b^	7.59 ± 0.35^c^
	PG concentration (%)			
	0	7.17 ± 0.14^c^	7.29 ± 0.19^c^	7.29 ± 0.16^c^
	0.25	7.53 ± 0.16^b^	7.61 ± 0.17^b^	7.77 ± 0.18^b^
	0.50	8.05 ± 0.16^a^	8.04 ± 0.17^a^	8.17 ± 0.19^a^

*Note:* Different letters in each column (treatments, i.e., MTGase or PG levels) indicate a significant difference at 95% level.

## 4 Conclusion

Dietary patterns among Iranian people indicate excessive dietary fat intake. The high fat consumption, particularly SFAs, appear to increase the risk of obesity, coronary heart disease, and developing certain types of cancer. Ultrafiltrated Iranian white cheese is one of the most important cheeses produced in Iran, widely consumed as a breakfast cheese. This cheese contains high amount of fat, comprise > 40% of cheese total solids. However, development of low‐fat cheese is challenging because fat plays a crucial role in the cheese quality, particularly its texture and taste. One of the most effective ways to repress these defects is to increase the moisture content of low‐fat cheese to establish a M:P ratio equal to or more than its full‐fat complement. Several researches revealed that addition of gums and MTGase treatment of cheese milk (alone or in combination) increases the water holding capacity of the low‐fat cheeses. Consequently, the aim of this study was to improve the quality parameters of low‐fat UF cheese by using PG and MTGase. Our results revealed that incorporation of both PG and MTGase increased the cheese moisture and M:P content and created a porous structure through proper formation of three‐dimensional casein network. Our findings show that the optimized low‐fat cheese sample (containing 0.5% PG and 0.5 U MTGase) had total acceptability, fatty acid composition, and microstructure characteristics similar to the full‐fat control. Therefore, by using a proper amount of PG and MTGase in the cheese making, a healthy low‐fat UF Iranian white cheese with an acceptable quality could be produced.

## Author Contributions

**Seyedeh Ameneh Habibi:** conceptualization (equal), formal analysis (supporting), methodology (equal), resources (equal), writing – original draft (lead). **Hossein Jooyandeh:** conceptualization (lead), data curation (lead), formal analysis (lead), investigation (lead), methodology (equal), project administration (lead), resources (equal), software (lead), supervision (lead), validation (lead), visualization (lead), writing – review and editing (lead).

## Ethics Statement

The authors have nothing to report.

## Conflicts of Interest

The authors declare no conflicts of interest.

## Data Availability

Data will be made available on request.

## References

[fsn34551-bib-0001] Abou‐Soliman, N. H. I. , S. Awad , and M. I. El‐Sayed . 2020. “The Impact of Microbial Transglutaminase on the Quality and Antioxidant Activity of Camel‐Milk Soft Cheese.” Food and Nutrition Sciences 11, no. 3: 153–171. 10.4236/fns.2020.113012.

[fsn34551-bib-0002] Aires da Silva, D. , G. Cristine Melo Aires , and R. da Silva Pena . 2021. “Gums‐characteristics and applications in the food industry.” In Innovation in the Food Sector Through the Valorization of Food and Agro‐Food By‐Products, edited by A. Novo de barros and I. Gouvinhas . Norderstedt, Deutschland: BOD – Books on Demand. 10.5772/intechopen.95078.

[fsn34551-bib-0003] Akbari, M. , M. H. Eskandari , and Z. Davoudi . 2019. “Application and Functions of Fat Replacers in Low‐Fat Ice Cream: A Review.” Trends in Food Science & Technology 86: 34–40. 10.1016/j.tifs.2019.02.036.

[fsn34551-bib-0004] AOAC . 2000. Official Methods of Analysis. 17th ed. Gaithersburg, MD: Association of Official Analytical Chemists.

[fsn34551-bib-0005] Beirami, F. , M. Hojjati , and H. Jooyandeh . 2021. “The Effect of Microbial Transglutaminase Enzyme and Persian Gum on the Characteristics of Traditional Kefir Drink.” International Dairy Journal 112: 104843. 10.1016/j.idairyj.2020.104843.

[fsn34551-bib-0006] Cadavid, A. M. , L. Bohigas , M. Toldrà , C. Carretero , D. Parés , and E. Saguer . 2020. “Improving Quark‐Type Cheese Yield and Quality by Treating Semi‐Skimmed Cow Milk With Microbial Transglutaminase.” LWT‐Food Science and Technology 131: 109756. 10.1016/j.lwt.2020.109756.

[fsn34551-bib-0007] Dabestani, M. , R. Kadkhodaee , G. O. Phillips , and S. Abbasi . 2018. “Persian Gum: A Comprehensive Review on Its Physicochemical and Functional Properties.” Food Hydrocolloids 78: 92–99. 10.1016/j.foodhyd.2017.06.006.

[fsn34551-bib-0008] Danesh, E. , M. Goudarzi , and H. Jooyandeh . 2018. “Transglutaminase‐Mediated Incorporation of Whey Protein as Fat Replacer Into the Formulation of Reduced‐Fat Iranian White Cheese: Physicochemical, Rheological and Microstructural Characterization.” Journal of Food Measurement and Characterization 12, no. 4: 2416–2425. 10.1007/s11694-018-9858-5.

[fsn34551-bib-0009] Eljagmani, S. , E. M. Altuner , and F. Yildiz . 2020. “Effect of Storage Temperature on the Chemical and Microbiological Properties of White Cheese From Kastamonu, Turkey.” Cogent Food & Agriculture 6, no. 1: 1829270. 10.1080/23311932.2020.1829270.

[fsn34551-bib-0010] Fox, P. F. , T. P. Guinee , T. M. Cogan , and P. L. H. McSweeney . 2017. Fundamentals of Cheese Science. 2nd ed, 1–28. Heidelberg, Germany: Springer. 10.1007/978-1-4899-7681-9.

[fsn34551-bib-0011] Fox, P. F. , P. L. H. McSweeney , T. M. Cogan , and T. P. Guinee . 2004. Cheese Chemistry, Physics and Microbiology. Vol. 1. 3rd ed. London, UK: Elsevier.

[fsn34551-bib-0012] Jooyandeh, H. 2024. Application of Enzymes in Dairy Products. 2nd ed. Mollasani, Iran: Khuzestan Agricultural Sciences and Natural Resources University Press.

[fsn34551-bib-0013] Jooyandeh, H. , and B. Alizadeh Behbahani . 2024. “Development of a Probiotic Low‐Fat Set Yogurt Containing Concentrated Sweet Pepper Extract.” Food Science & Nutrition 12, no. 7: 4656–4666. 10.1002/fsn3.4114. 39055224 PMC11266907

[fsn34551-bib-0014] Jooyandeh, H. , M. Goudarzi , H. Rostamabadi , and M. Hojjati . 2017. “Effect of Persian and Almond Gums as Fat Replacers on the Physicochemical, Rheological, and Microstructural Attributes of Low‐Fat Iranian White Cheese.” Food Science & Nutrition 5, no. 3: 669–677. 10.1002/fsn3.446. 28572956 PMC5448388

[fsn34551-bib-0015] Jooyandeh, H. , A. Kaur , and K. S. Minhas . 2009. “Lipases in Dairy Industry.” Journal of Food Science and Technology 46, no. 3: 181–189.

[fsn34551-bib-0016] Jooyandeh, H. , and K. S. Minhas . 2009. “Effect of Addition of Fermented Whey Protein Concentrate on Cheese Yield and Fat and Protein Recoveries of Feta Cheese.” Journal of Food Science and Technology 46, no. 3: 221–224.

[fsn34551-bib-0017] Jooyandeh, H. , S. Momenzadeh , B. Alizadeh Behbahani , and H. Barzegar . 2022. “Effect of *Malva neglecta* and Lactulose on Survival of *Lactobacillus fermentum* and Textural Properties of Symbiotic Stirred Yogurt.” Journal of Food Science and Technology 60: 1136–1143. 10.1007/s13197-023-05667-6. PMC9998791 36908339

[fsn34551-bib-0018] Jooyandeh, H. , M. Noshad , and R. A. Khamirian . 2018. “Modeling of Ultrasound‐Assisted Extraction, Characterization and In Vitro Pharmacological Potential of Polysaccharides From *Vaccinium arctostaphylos* L.” International Journal of Biological Macromolecules 107, no. part A: 938–948. 10.1016/j.ijbiomac.2017.09.077. 28939523

[fsn34551-bib-0019] Jooyandeh, H. , S. Saffari Samani , B. Alizadeh Behbahani , and M. Noshad . 2022. “Effect of Transglutaminase and Buffalo Milk Incorporation on Textural Parameters and Starter Cultures Viability of Strained Yogurt.” Journal of Food and Bioprocess Engineering 5, no. 2: 195–202. 10.22059/JFABE.2023.351980.1133.

[fsn34551-bib-0020] Kawęcka, A. , I. Radkowska , and J. Sikora . 2020. “Concentrations of Selected Bioactive Components in Traditional Cheeses Made From Goat's, Cow's and Sheep's Milk.” Journal of Elementology 25: 431–442. 10.5601/jelem.2019.24.3.1907.

[fsn34551-bib-0021] Kouravand, F. , H. Jooyandeh , H. Barzegar , and M. Hojjati . 2020. “Mechanical, Barrier and Structural Properties of Whey Protein Isolate‐Based Films Treated by Microbial Transglutaminase.” Journal of Microbiology, Biotechnology and Food Sciences 9, no. 5: 960–964. 10.15414/jmbfs.2020.9.5.960-964.

[fsn34551-bib-0022] Monsalve‐Atencio, R. , K. Sanchez‐Soto , J. Chica , J. A. C. Echavarría , and O. Vega‐Castro . 2022. “Interaction Between Phospholipase and Transglutaminase in the Production of Semi‐Soft Fresh Cheese and Its Effect on the Yield, Composition, Microstructure and Textural Properties.” LWT‐Food Science and Technology 154: 112722. 10.1016/j.lwt.2021.112722.

[fsn34551-bib-0023] Mosallaie, F. , H. Jooyandeh , M. Hojjati , and A. Fazlara . 2020. “Biological Reduction of Aflatoxin B_1_ in Yogurt by Probiotic Strains of *Lactobacillus acidophilus* and *Lactobacillus rhamnosus* .” Food Science and Biotechnology 29, no. 6: 793–803. 10.1007/s10068-019-00722-5. 32523789 PMC7256161

[fsn34551-bib-0024] Muhammad, A. S. , A. A. Abdulqader , W. Al Ansi , et al. 2021. “Current Industrial Applications of Microbial Transglutaminase: A Review.” International Journal of Advanced Engineering, Management and Science 7, no. 3: 81–92. 10.22161/ijaems.73.11.

[fsn34551-bib-0025] Murtaza, M. S. , A. Sameen , N. Huma , and F. Hussain . 2017. “Influence of Hydrocolloid Gums on Textural, Functional and Sensory Properties of Low Fat Cheddar Cheese From Buffalo Milk.” Pakistan Journal of Zoology 49, no. 1: 27–34. 10.17582/journal.pjz/2017.49.1.27.34.

[fsn34551-bib-0026] Nosrati, G. , H. Jooyandeh , M. Hojjati , and M. Noshad . 2023. “Study on the Physicochemical Properties of Synbiotic UF‐Cheese Containing Demineralized Ultrafiltrated Whey Powder and Lactulose During Storage Period.” Iranian Journal of Food Science and Technology 20, no. 139: 149–164. 10.22034/FSCT.20.139.164.

[fsn34551-bib-0027] Ozer, B. , H. A. Kirmaci , S. Oztekin , A. Hayaloglu , and M. Atamer . 2007. “Incorporation of Microbial Transglutaminase Into Non‐fat Yogurt Production.” International Dairy Journal 17: 199–207. 10.1016/j.idairyj.2006.02.007.

[fsn34551-bib-0028] Paszczyk, B. , and J. Łuczyńska . 2020. “The Comparison of Fatty Acid Composition and Lipid Quality Indices in Hard Cow, Sheep, and Goat Cheeses.” Food 9, no. 11: 1667. 10.3390/foods9111667. PMC7696827 33203107

[fsn34551-bib-0029] Portaghi, J. , A. Heshmati , M. Taheri , E. Ahmadi , and A. M. Khaneghah . 2023. “Effect of Basil Seed and Xanthan Gum on Physicochemical, Textural, and Sensory Characteristics of Low‐Fat Cream Cheese.” Food Science & Nutrition 11, no. 10: 6060–6072. 10.1002/fsn3.3542. 37823144 PMC10563744

[fsn34551-bib-0030] Rahimi, J. , A. Khosrowshahi , A. Madadlou , and S. Aziznia . 2007. “Texture of Low‐Fat Iranian White Cheese as Influenced by Gum Tragacanth as a Fat Replacer.” Journal of Dairy Science 90, no. 9: 4058–4070. 10.3168/jds.2007-0121. 17699022

[fsn34551-bib-0031] Razeghi, F. , and S. Yazdanpanah . 2020. “Effects of Free and Encapsulated Transglutaminase on the Physicochemical, Textural, Microbial, Sensorial, and Microstructural Properties of White Cheese.” Food Science & Nutrition 8, no. 7: 3750–3758. 10.1002/fsn3.1663. 32724637 PMC7382158

[fsn34551-bib-0032] Romeih, E. A. , A. Michaelidou , C. G. Biliaderis , and G. K. Zerfiridis . 2002. “Low‐Fat White‐Brined Cheese Made From Bovine Milk and Two Commercial Fat Mimetics: Chemical, Physical and Sensory Attributes.” International Dairy Journal 12, no. 6: 525–540. 10.1016/S0958-6946(02)00043-2.

[fsn34551-bib-0033] Rostamabadi, H. , H. Jooyandeh , and M. Hojjati . 2017. “Optimization of Physicochemical, Sensorial and Color Properties of Ultrafiltrated Low‐Fat Iranian White Cheese Containing Fat Replacers by Response Surface Methodology.” Iranian Journal of Food Science and Technology 14, no. 63: 91–106. https://fsct.modares.ac.ir/article‐7‐2151‐fa.pdf.

[fsn34551-bib-0034] Saffari Samani, E. , H. Jooyandeh , and B. Alizadeh Behbahani . 2023. “The Impact of Zedo Gum Based Edible Coating Containing *Zataria Multiflora* Boiss Essential Oil on the Quality Enhancement and Shelf Life Improvement of Fresh Buffalo Meat.” Journal of Food Measurement and Characterization 17: 2663–2675. 10.1007/s11694-023-01811-0.

[fsn34551-bib-0035] Salunke, P. , C. Marella , J. K. Amamcharla , K. Muthukumarappan , and L. E. Metzger . 2022a. “Use of Micellar Casein Concentrate and Milk Protein Concentrate Treated With Transglutaminase in Imitation Cheese Products‐Unmelted Texture.” Journal of Dairy Science 105, no. 10: 7891–7903. 10.3168/jds.2022-21852. 36055836

[fsn34551-bib-0036] Salunke, P. , C. Marella , J. K. Amamcharla , K. Muthukumarappan , and L. E. Metzger . 2022b. “Use of Micellar Casein Concentrate and Milk Protein Concentrate Treated With Transglutaminase in Imitation Cheese Products‐Melt and Stretch Properties.” Journal of Dairy Science 105, no. 10: 7904–7916. 10.3168/jds.2022-22253. 36055846

[fsn34551-bib-0037] Seyed‐Moslemi, S. A. , J. Hesari , S. H. Peighambardoust , and S. J. Peighambardoust . 2021. “Effect of Microbial Lipase and Transglutaminase on the Textural, Physicochemical, and Microbial Parameters of Fresh Quark Cheese.” Journal of Dairy Science 104, no. 7: 7489–7499. 10.3168/jds.2020-19781. 33985784

[fsn34551-bib-0038] Sharif, N. , I. Falcó , A. Martínez‐Abad , G. Sánchez , A. López‐Rubio , and M. J. Fabra . 2021. “On the Use of Persian Gum for the Development of Antiviral Edible Coatings Against Murine Norovirus of Interest in Blueberries.” Polymers 13, no. 2: 224. 10.3390/polym13020224. 33440825 PMC7827901

[fsn34551-bib-0039] Temiz, H. , and E. Çakmak . 2018. “The Effect of Microbial Transglutaminase on Probiotic Fermented Milk Produced Using a Mixture of Bovine Milk and Soy Drink.” International Journal of Dairy Technology 71: 906–920. 10.1111/1471-0307.12521.

[fsn34551-bib-0040] Torabi, F. , H. Jooyandeh , and M. Noshad . 2021. “Evaluation of Physicochemical, Rheological, Microstructural, and Microbial Characteristics of Synbiotic Ultrafiltrated White Cheese Treated With Transglutaminase.” Journal of Food Processing & Preservation 45, no. 6: 15572. 10.1111/jfpp.15572.

[fsn34551-bib-0041] Vasić, K. , Ž. Knez , and M. Leitgeb . 2023. “Transglutaminase in Foods and Biotechnology.” International Journal of Molecular Sciences 24, no. 15: 12402. 10.3390/ijms241512402. 37569776 PMC10419021

[fsn34551-bib-0042] Warsono, E. K. , L. Evlyn , M. Janice , and F. P. Rizfi . 2021. “Recent Advances in the Use of Transglutaminase in Cheese Production.” ASEAN Journal on Science & Technology for Development 38, no. 2: 83–88. 10.29037/ajstd.675.

[fsn34551-bib-0043] Weaver, C. M. 2021. “Dairy Matrix: Is the Whole Greater Than the Sum of the Parts?” Nutrition Reviews 79, no. 2: 4–15. 10.1093/nutrit/nuab081. PMC8653934 34879148

[fsn34551-bib-0044] Wen, P. , Y. Zhu , J. Luo , et al. 2021. “Effect of Anthocyanin‐Absorbed Whey Protein Microgels on Physicochemical and Textural Properties of Reduced‐Fat Cheddar Cheese.” Journal of Dairy Science 104, no. 1: 228–242. 10.3168/jds.2020-18450. 33189294

[fsn34551-bib-0045] Zare‐Bavani, M. R. , M. Rahmati‐Joneidabad , and H. Jooyandeh . 2024. “Gum Tragacanth, A Novel Edible Coating, Maintains Biochemical Quality, Antioxidant Capacity, and Storage Life in Bell Pepper Fruits.” Food Science & Nutrition 12, no. 6: 3935–3948. 10.1002/fsn3.4052. 38873491 PMC11167171

